# The Bezold–Jarisch Reflex Following an Endoscopic Endonasal Resection of an Intraosseous Clival Myxoma: A Case Report

**DOI:** 10.1155/cria/3336045

**Published:** 2025-02-20

**Authors:** Abigail Peterson, Omar Hussain, Nathan Zwagerman, Harvey Woehlck

**Affiliations:** ^1^Department of Neurosurgery, Medical College of Wisconsin, Milwaukee, Wisconsin, USA; ^2^Department of Anesthesiology, Medical College of Wisconsin, Milwaukee, Wisconsin, USA

**Keywords:** Bezold–Jarisch reflex, case report, clival tumor, endoscopic endonasal approach, intraosseous myxoma

## Abstract

In certain contexts, young and healthy patients with a strong heart and a history of vasovagal syncope are at increased risk of cardiac arrest. An increase in vagal tone results in the normal maintenance of arterial pressure shifting into parasympathetic activation and sympathetic suppression, amplifying afferent mechanoreceptors and, in rare instances, inducing asystole. We report the case of a 17-year-old patient with a past medical history of syncope who went into asystole while maintaining consciousness and protecting his airway when recovering from anesthesia in the postanesthesia care unit (PACU) following endoscopic endonasal resection of an intraosseous clival myxoma. Chest compressions were initiated and epinephrine was administered, allowing for return of spontaneous circulation to be quickly achieved. While being transferred to the intensive care unit, the patient's heart rate dropped to 20 bpm while sitting in Fowler's position, causing vasovagal syncope which was then resolved by laying the patient supine and 0.8 mg of glycopyrrolate administration. The altered sympathetic to parasympathetic tone resulting in asystole within this case and cardiac beta-agonist stimulation by epinephrine injection provide evidence that the Bezold–Jarisch reflex occurred. This case suggests that the intracranial internal carotid arteries can potentially display similar mechanical sensitivity as the carotid sinus and questions the validity of electrocardiogram readings during this reflex, as the patient remained conscious while in asystole.

## 1. Introduction

Post-operative cardiac arrest associated with neurosurgical procedures often yields poor patient outcomes. Circulatory response changes occurring during regional anesthesia or arterial compression are controlled by various baroreceptors and afferent cardiac nerves. The normal physiological regulation of the heart's sinus rhythm is complex, responding to various intrinsic and extrinsic signals. The central nervous system (CNS) plays a large role in extrinsic regulation, largely being controlled by a vasomotor complex in the medulla oblongata [1]. The medulla oblongata primarily activates pathways in the autonomic nervous system, affecting blood vessel constriction and dilation. The aorta and carotid arteries also engage with each other to control the sympathetic outflow of blood to the periphery and coordinate responses to stress [2]. The carotid sinus can be hypersensitive to stimulation, often resulting in bradycardia, vasodilation, and hypotension [3]. When responding to hypotension under normal physiologic conditions, baroreceptors in the left ventricular (LV) wall trigger systemic vasoconstriction and subsequent tachycardia.

Cardiovascular changes occurring in response to postural changes are also coordinated by baroreceptors. When transitioning from lying supine to sitting upright (Fowler's position), gravity forces redistribution of blood by decreasing venous return to the heart and decreasing preload. Venous blood flow is instead distributed to the legs. To compensate, the baroreceptor reflex becomes activated to decrease vagal tone, decreasing compliance of venous circulation to the legs and lowering blood pressure.

**Figure 1:**
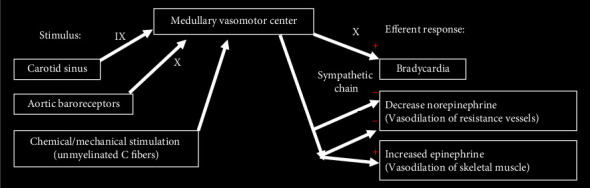
Neurally-mediated syncope via the vagus nerve and sympathetic afferents. + stimulates, − inhibits.

Trauma or physiologic failure to autoregulate blood pressure can result in vasovagal syncope, characterized by decreased cardiac output and transient loss of consciousness [4]. Internal carotid sinuses modulate the vagus nerve by regulating baroreceptor afferents. Manipulation of these arteries can simulate an elevation in systolic blood pressure, triggering subsequent parasympathetic stimulation in which the vagus nerve slows heart rate and decreases blood pressure in what is often referred to as “collapse firing” [1]. This vasodepressive reflex, the Bezold–Jarisch reflex, can be visualized in Figure 1.

We present a unique case in which a patient with a past medical history of vasovagal syncope underwent a procedure in which the sympathetic efferent cardiac chains and compensatory mechanisms were inhibited, resulting in postoperative asystole. We will discuss relevant physiological mechanisms that explain how this case is consistent with the Bezold–Jarisch reflex and examine possible management strategies.

## 2. Case Report

**Figure 2:**
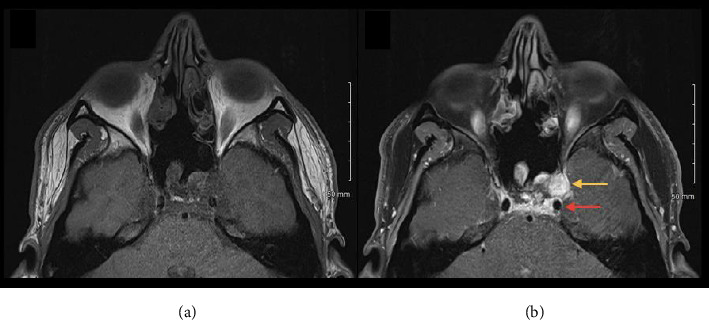
MRI brain (a) pre- and (b) postcontrast demonstrating a contrast-enhancing lesion (yellow arrow) medial to the left temporal lobe within the cavernous sinus, and anterior to the internal carotid artery (red arrow).

A teenage male with a history of large intraosseous clival myxoma, which was previously resected through an endoscopic endonasal approach, was found to have continued growth of the residual lesion around the anterior portion of the left cavernous sinus on surveillance imaging at 18 months postoperatively (Figure 2). He then underwent further resection through a redo endoscopic endonasal approach. Before surgery began, the mucosa in his nares was injected with lidocaine with epinephrine to aid in pain control and hemostasis. Intraoperatively, dissection into the cavernous sinus was performed along with skeletonization and subsequent exposure of the internal carotid artery (ICA) to ensure complete resection of the intracavernous lesion. While blunt dissection and tumor resection took place over and around the ICA, there were no intraoperative complications or heart rate variability. Given his stable vitals and healthy physiology, he required no pressors, and received minimal amounts of intravenous fluids.

Following the surgery, the patient was transported to the postoperative anesthesia care unit (PACU) without complication, awake and alert, and hemodynamically stable with vital signs within normal limits. When recovering from anesthesia in the PACU, the patient's heart rate trended down to asystole for approximately 90–120 s. During this time, he was awake but increasingly lethargic and protecting his airway. A pulse check was done by the PACU team, which was unsuccessful in identifying any pulse, and chest compressions were initiated for less than 30 s. Shortly afterward, 10 mcg of epinephrine was administered, resulting in a return of spontaneous circulation (ROSC) to be quickly achieved. He was monitored in the PACU closely with normal vital signs without any electrolyte or electrocardiogram abnormalities while being transferred to the intensive care unit (ICU). While sitting upright in his bed, the patient's heart rate dropped to 20 bpm and he experienced dizziness, which was then resolved by lying supine and administration of 0.8 mg glycopyrrolate.

Upon arrival to the ICU, a trial of sitting upright resulted in hypotension with SBP of 70 mmHg along with symptoms of presyncope, both of which resolved with lying flat and the initiation of low-dose epinephrine drip. The patient continued to do well with normalization of his activity while on the low-dose epinephrine drip. He was started on pseudoephedrine 60 mg every 6 h on postoperative day 0 and was eventually weaned off the epinephrine by that evening. No other episodes occurred, he remained hemodynamically stable without intravenous pressors, and he was discharged home on postoperative day 1 with the oral pseudoephedrine. The patient was seen by cardiology as an outpatient and was diagnosed with high vagal tone after a normal echocardiogram was performed.

## 3. Discussion

We believe this to be the first case of the Bezold–Jarisch reflex occurring following an endoscopic endonasal neurosurgical procedure. The described postoperative asystole is consistent with the Bezold–Jarisch reflex, hypothesized to be triggered by the manipulation of the intracavernous portion of the ICA during the endoscopic endonasal resection of the interosseous clival myxoma. Minor episodes of carotid obstruction were likely during the procedure in this case due to the proximity of the clivus myxoma to the left ICA. This ICA-stimulated reflex is theoretical as no literature mentions the presence of baroreceptors within the ICA as it enters the skull. It is also possible that the setting of the nasal surgery could have elicited the Bezold–Jarisch reflex by stimulating trigeminal afferent fibers which could have inhibited the cardiac vascular system [5].

The preoperative injection of epinephrine for hemostasis, along with the postoperative administration given to achieve ROSC, established conducive conditions for the manifestation of the Bezold–Jarisch reflex. Epinephrine, a potent agonist of cardiac alpha and beta receptors, increases systolic blood pressure and myocardial oxygen consumption [6]. In this case, epinephrine most likely elicited vasoconstriction and facilitated ROSC. This corroborates the hypothesis that the Bezold–Jarisch reflex induced bradycardia in our patient by blocking sympathetic cardiac activity. Essentially, this reflex disconnected the body from the cranial sympathetic nervous system and eliminated meaningful brainstem-mediated vasoconstriction, which was subsequently stimulated by epinephrine.

Our patient was predisposed to the Bezold–Jarisch reflex, having a high baseline vagal tone and previous episodes of vasovagal syncope with increased activity. Also, following his return to ROSC after epinephrine injection and chest compressions, the patient was sitting in an upright position, causing venous pooling and transient intravascular hypovolemia that could have exacerbated a vasodepressor response [7].

A review of articles discussing the Bezold–Jarisch reflex provided little data regarding the outcomes of resections of intraosseous myxomas originating in the clivus, given that only two other cases have been reported [8, 9]. However, cases involving neural stimulation causing this reflex resulted in similar clinical outcomes. In one report, Dilip Chand Raja et al. observed direct stimulation of the right vagus nerve resulting in sudden bradycardia, hypotension, and asystole in a 17-year-old patient following a corpectomy of C5, C6, and C7 [10]. The reported resolution of dynamic thoracic inlet compression, which initially caused the Bezold–Jarisch reflex, was resolved when the patient was moved from the prone to the supine position intraoperatively. Another case described the Bezold–Jarisch Reflex during a Deep-Brain stimulation (DBS) procedure within a patient that also had a history of vasovagal syncope, administration of epinephrine, and was resolved with glycopyrrolate, experiencing similar stimuli as in this case [5].

Clinical management of patients undergoing neurosurgical procedures can improve by clinicians becoming aware of the factors predisposing patients to the Bezold–Jarisch reflex. Patients with a medical history of high vagal tone are at risk of this reflex and should be monitored closely perioperatively. During surgical procedures, the mechanical stretch of baroreceptors in the carotid arteries and its branches should also be regarded. Additionally, avoiding positioning patients from sitting in an upward position could prevent severe bradycardia by reducing cardiac hypovolemia, which can be exacerbated in a patient who has received minimal intravenous fluids while limiting their oral intake prior to surgery [9]. Administration of epinephrine and glycopyrrolate are interventions that can resolve severe bradycardia, stimulating cardiac alpha and beta receptors and blocking muscarinic effects caused by cholinesterase, respectively. While normal doses of epinephrine can adequately prevent bradycardia, 0.8 mg of glycopyrrolate is the recommended dosage given its' unique pharmacokinetic properties. In our experience, underdosing with glycopyrrolate can either exacerbate or overcompensate for a patient's bradycardia. If medications are not immediately available, a simply passive leg raise would theoretically transiently increase cardiac preload, leading to a mechanical stretch of the right atrium, leading to an increase in the heart rate.

## 4. Conclusions

This case report highlights the importance of careful patient management to prevent perioperative bradycardia and hypotension. Patients with a strong heart and a history of high baseline vagal tone are at risk of the Bezold–Jarisch reflex occurring during an endoscopic endonasal resection. Avoiding hypovolemia and the administration of epinephrine and glycopyrrolate are appropriate treatment interventions to prevent bradycardia, apnea, and hypotension.

## Data Availability

The data used to support the findings of this study are included within the article.

## References

[1] Kinsella S. M., Tuckey J. P. (2001). Perioperative Bradycardia and Asystole: Relationship to Vasovagal Syncope and the Bezold–Jarisch Reflex. *British Journal of Anaesthesia*.

[2] Karemaker J. M. (2022). The Multibranched Nerve: Vagal Function Beyond Heart Rate Variability. *Biological Psychology*.

[3] Kharsa A., Wadhwa R. (2022). *Carotid Sinus Hypersensitivity*.

[4] Jeanmonod R., Sahni D., Silberman M. (2023). *Vasovagal Episode*.

[5] Nguyen H. S., Woehlck H., Pahapill P. (2016). An Unusual Case of Asystole Occurring During Deep Brain Stimulation Surgery. *Case Reports in Neurological Medicine*.

[6] Morales-Cané I., Valverde-León M. D., Rodríguez-Borrego M. A. (2016). Epinephrine in Cardiac Arrest: Systematic Review and Meta-Analysis. *Revista Latino-Americana de Enfermagem*.

[7] Goila A., Garg R., Pawar M. (2011). Catastrophic Complication-Bezold-Jarisch Reflex: Case Series. *Indian Journal of Anaesthesia*.

[8] Weng J. C., Song L. R., Li D. (2019). Surgical Management and Prognostic Factors for Primary Intracranial Myxoma: A Single-Institute Experience With a Systematic Review. *Journal of Neurosurgery*.

[9] Erdem Y., Koktekir E., Bayar M. A., Yilmaz A., Caydere M. (2012). Characterization of an Intracranial Neurothekeoma: Case Report. *Turkish Neurosurgery*.

[10] Dilip Chand Raja S., Rajasekaran S., Sri Vijayanand K. S., Shetty A. P., Kanna R. M. (2020). Bezold-Jarisch Reflex Causing Bradycardia and Hypotension in a Case of Severe Dystrophic Cervical Kyphotic Deformity: A Case Report and Review of Literature. *European Spine Journal*.

